# Identification of putative TAL effector targets of the citrus canker pathogens shows functional convergence underlying disease development and defense response

**DOI:** 10.1186/1471-2164-15-157

**Published:** 2014-02-25

**Authors:** Andre LA Pereira, Marcelo F Carazzolle, Valeria Y Abe, Maria LP de Oliveira, Mariane N Domingues, Jaqueline C Silva, Raul A Cernadas, Celso E Benedetti

**Affiliations:** 1 Laboratório Nacional de Biociências, Centro Nacional de Pesquisa em Energia e Materiais, R. Giuseppe Máximo Scolfaro 10000, Campinas, SP 13083-970, Brazil; 2 Departamento de Genética, Evolução e Bioagentes, Instituto de Biologia, Universidade Estadual de Campinas, Campinas, SP 13083-970, Brazil; 3 Present address: U.S. Department of Agriculture, U.S. Horticultural Research Laboratory, 2001 South Rock Road, Fort Pierce, FL 34945, USA; 4 Present address: Department of Plant Pathology and Plant-Microbe Biology, Cornell University, Ithaca, NY 14853, USA

**Keywords:** TAL effectors, PthA, PthC, *Xanthomonas citri*, *Xanthomonas aurantifolii*, Citrus canker, Target genes

## Abstract

**Background:**

Transcriptional activator-like (TAL) effectors, formerly known as the AvrBs3/PthA protein family, are DNA-binding effectors broadly found in *Xanthomonas spp.* that transactivate host genes upon injection via the bacterial type three-secretion system. Biologically relevant targets of TAL effectors, i.e. host genes whose induction is vital to establish a compatible interaction, have been reported for xanthomonads that colonize rice and pepper; however, citrus genes modulated by the TAL effectors PthA“s” and PthC“s” of the citrus canker bacteria *Xanthomonas citri* (Xc) and *Xanthomonas aurantifolii* pathotype C (XaC), respectively, are poorly characterized. Of particular interest, XaC causes canker disease in its host lemon (*Citrus aurantifolia*), but triggers a defense response in sweet orange.

**Results:**

Based on, 1) the TAL effector-DNA binding code, 2) gene expression data of Xc and XaC-infiltrated sweet orange leaves, and 3) citrus hypocotyls transformed with PthA2, PthA4 or PthC1, we have identified a collection of *Citrus sinensis* genes potentially targeted by Xc and XaC TAL effectors. Our results suggest that similar with other strains of *Xanthomonas* TAL effectors, PthA2 and PthA4, and PthC1 to some extent, functionally converge. In particular, towards induction of genes involved in the auxin and gibberellin synthesis and response, cell division, and defense response. We also present evidence indicating that the TAL effectors act as transcriptional repressors and that the best scoring predicted DNA targets of PthA“s” and PthC“s” in citrus promoters predominantly overlap with or localize near to TATA boxes of core promoters, supporting the idea that TAL effectors interact with the host basal transcriptional machinery to recruit the RNA pol II and start transcription.

**Conclusions:**

The identification of PthA“s” and PthC“s” targets, such as the *LOB* (*LATERAL ORGAN BOUNDARY*) and *CCNBS* genes that we report here, is key for the understanding of the canker symptoms development during host susceptibility, or the defenses of sweet orange against the canker bacteria. We have narrowed down candidate targets to a few, which pointed out the host metabolic pathways explored by the pathogens.

## Background

Transcriptional activator-like (TAL) effectors of *Xanthomonas spp.* had been featured as central determinants of both bacterial pathogenicity and avirulence in numerous plant species or cultivars [1-6]. However, it was not until recently that the biochemical function of TAL effectors as transcriptional regulators was discovered [6-13].

The tridimensional structure of TAL effectors showed that these proteins are distinct from any other bacterial effectors that are targeted to the interior of the host cell by the type-III secretion system [14-17]. TAL effectors striking signature is made by the polymorphisms in positions 12-13^th^ of the 33-34 amino acids tandem repeats, referred as repeat-variable diresidues (RVDs), which comprise the DNA-binding domain of the effector [12,13]. The consecutive repeats wrap around the DNA double helix, accommodating the RVDs adjacent to the target DNA bases in a one-to-one RVD-DNA base fashion, which are stabilized by hydrogen bonds and/or Wan-der Waals forces between the 13^th^ RVD residue and the DNA base [15-17]. These TAL effector-targeted sequences have been initially called UPT (up-regulated by TAL effector) boxes, and later more broadly, Effector Binding Elements (EBEs) [6,9]. The discovery of the TAL effector code has provided an invaluable tool for genome engineering by user-designed TAL effectors fused to catalytic domains, or designer TAL effectors to activate gene expression and explore their function during bacterial infection processes [18-20].

Based on host-range, *Xanthomonas citri* strains belong into different pathotypes being the Asian group A the most aggressive that exhibit wide-host range. Strains from groups B and C form a phylogenetically distinct clade originated in South America that exhibit limited host range [4,21]. The TAL effectors identified in *X. citri* strains were designated PthA“s”, PthB“s” and PthC“s”, and despite they are inherent in pathogenicity, the corresponding host gene targets remain uncharacterized [2,4,21-23]. Interestingly, a pioneering study showed that pathotypes A, B and C carry at least one isofunctional PthA, PthB or PthC effector of 17.5 repeat domains, which is essentially required to elicit hyperplastic canker lesions on citrus [4,21]. On the other hand, none of the TAL effectors from the limited-host-range strains (pathotypes B and C) was able to trigger the hypersensitive response (HR) observed in grapefruit plants when expressed heterologously in other strain, suggesting that TAL effectors from citrus canker pathogens do not limit host range but rather contribute to virulence associated functions [4,21].

Recent reports have focused on the computational-based prediction of EBEs in plant genomes to identify the putative host gene targets of TAL effectors [13,24,25]. For citrus, the *in silico* analyses to predict PthA“s” targets in sweet orange provided a set of candidates but additional experimental evidence of, e.g. gene expression, is still needed to validate such predictions [24]. Also, those studies did not include PthB“s” or PthC“s” effectors from the restricted host-range strains like *Xanthomonas aurantifolii* pathotype C (XaC), which in turn trigger a defense response in sweet oranges. Such analyses could provide molecular candidates that regulate the citrus defense response against *Xanthomonas spp.*[24,26]. In other pathosystems, the identification of TAL effector-induced genes of *X. vesicatoria* and *X. oryzae* has revealed novel virulence mechanisms of plant bacteria mediated by the targeted transcriptional induction of key regulators of host susceptibility [3,6,8,11]. Therefore, identification of TAL effector targeted factors that regulate citrus canker susceptibility is a milestone to understand and improve disease resistance.

Using microarray analyses, we have previously shown that the genes up-regulated by XaC in sweet orange leaves are involved in basal defense. In contrast, *X. citri* (Xc) induced genes associated with cell division and growth at the beginning of the infection process [26]. We also found that many of the rapidly Xc-induced genes, including cellulases, expansins and other cell-wall remodeling proteins, are co-regulated by auxin and gibberellin, hormones that are required for canker development [27] and control cell growth and expansion in other plant species [28]. TAL effectors not only play a central role as major determinants of host susceptibility, but are also capable of eliciting a resistance response when targeting HR-executor genes [3,6-8,29,30]. Based on these evidences, we hypothesized that TAL effectors from Xc and XaC are directly regulating the transcription of sweet orange genes involved in canker formation and defense response, respectively.

In this study, we present a combination of bioinformatics, microarray analyses, and molecular assays to identify sweet orange genes targeted by PthA2, PthA4 and PthC1 proteins. We show that the ectopic expression of PthA2, PthA4 or PthC1 in citrus epicotyls resulted in the up-regulation of a group of genes involved in auxin and gibberellin response, cell growth, and defense response. Our *in silico* studies using the TAL effector code, predicted many EBEs for the PthA“s” and/or PthC effectors in the promoter regions of genes induced in epicotyls expressing the corresponding TAL effector. Interestingly, we noticed that the EBEs overlap with, or localize close to TATA box elements of the promoters. In addition, despite the different RVD composition between PthA“s” and PthC“s”, our results indicate a targeting of functionally-related genes, which further support a model where TAL effectors display the functional convergence by selective evolution as general TATA-binding proteins [24,25]. Finally, we present experimental evidence suggesting that TAL effectors from citrus canker pathogens also function as transcriptional repressors.

## Results

### Transcriptional changes in sweet orange triggered by Xc TAL effectors

By extensive gene expression analyses, we had identified numerous genes up-regulated during the canker disease development of sweet orange leaves infiltrated with Xc [26]. To test whether any of those genes are direct targets of TAL effectors, we have undertaken two complementary approaches. First, we performed microarrays assays of orange leaves after bacterial infiltration in the presence or absence of the protein synthesis inhibitor, cycloheximide (Ch), a strategy that has early pinpointed AvrBs3 targets in pepper plants [10,31]. We found that many of the genes induced by Xc at 6 and/or 48 h after infiltration [26] are also induced by Xc in the presence of Ch (Additional file 1), thus indicating that Xc elicit major transcriptional reprograming independent of protein synthesis. Several of these differentially expressed genes are likely involved in terpene and gibberellin synthesis, ethylene production and signaling, cell-wall remodeling, cell division and defense responses (Additional file 1). In particular, we noticed that the ethylene synthesis pathway represented by orthologs of ACC synthase, ACC oxidase and AP2 factor genes, which play roles in cell wall softening [32-34], appears to be a primary mechanism elicited in the host after Xc sensing. On the other hand, defense response genes encoding chitinases, WRKY factors and pathogenesis-related (PR) proteins are also rapidly induced (Additional file 1).

**Figure 1:**
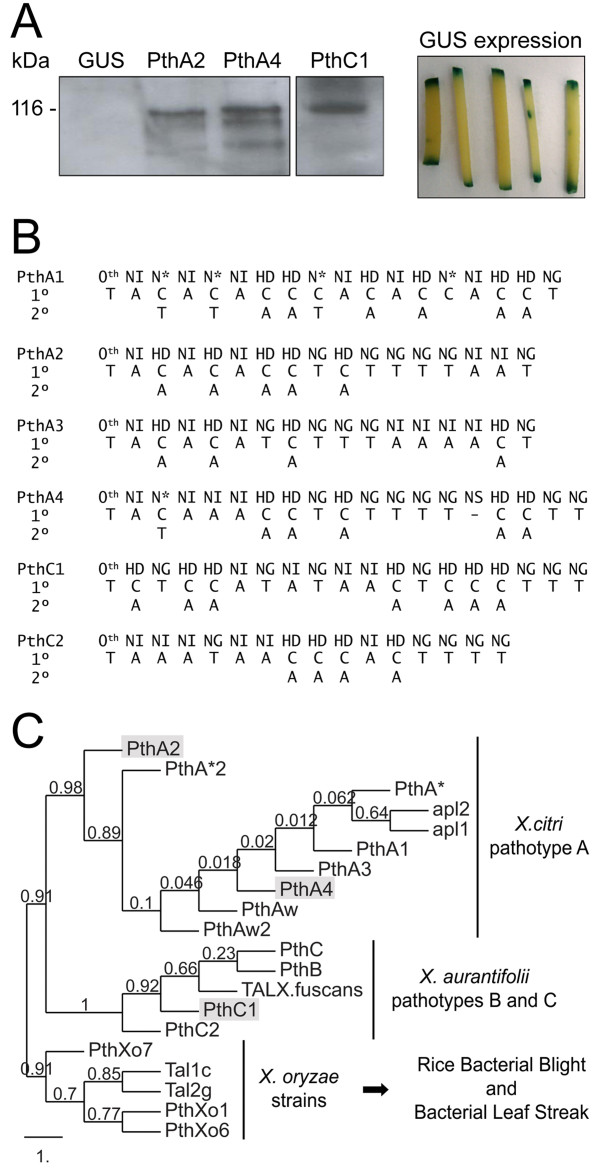
**Heterologous expression in citrus epicotyls, RVD sequences and phylogeny of TAL effectors PthA2, PthA4 and PthC1. (A)** Western blot of protein extracts from sweet orange epicotyls transfected with *A. tumefaciens* EHA105 carrying pBI121-35S::*pthA2* (PthA2), pBI121-35S::*pthA4* (PthA4), pBI121-35S::*pthC1* (PthC1), or the native plasmid pBI121-35S::*uidA* (GUS) for control of TAL effectors expression. Total protein from epicotyls expressing PthA2, PthA4 or, PthC1 proteins (~116 kDa) were separated by electrophoresis on 10% SDS-polyacrylamide gels, transferred to PVDF membranes, and detected with anti-PthA2 serum (left panel). The expression of *uidA* gene was assayed histochemically for β-glucuronidase (GUS) activity using 5-bromo-4-chloro-3-indolyl-β-D-glucuronic acid (X-Gluc) as substrate (right panel). **(B)** RVD sequence composition of *X. citri* isolate 306 TAL effectors PthA1, PthA2, PthA3 and PthA4, and PthC1 and PthC2 from *X. aurantifolii* ICMP 8435, aligned with the corresponding predicted DNA targets. According to the TAL effector code, only the first and second bases associated with higher frequency for each RVD are represented. **(C)** Phylogenetic tree of TAL effectors from different *Xanthomonas* strains that cause citrus canker disease and blight or leaf streak of rice. The maximum likelihood analysis was built with the PhyML tool using a bootstrapping procedure of 500 repetitions. Only the C-terminal domains (~278 residues) of TAL effectors from *Xanthomonas spp.* were used for the analysis. The four PthA“s” of Xc strain 306 belong into a group close to other TAL effectors from pathotype A strains; meanwhile, PthCs and PthBs from *X. aurantifolii* integrate a distinct group of pathotypes B and C strains, respectively. The tree is displayed with the TAL effectors from *X. oryzae* strains rooted as outgroup. Amino acid sequences were aligned using MUSCLE and analyzed on phylogenetic pipeline of *Phylogeny.fr*[41].

Second, we transiently expressed the Xc TAL effectors PthA2 and PthA4 in sweet orange epicotyls and compared the transcriptional changes relative to epicotyls transformed with GUS as a control (Figure 1A). We selected PthA2 and PthA4 because they form heterodimers and interact with several citrus proteins implicated in transcriptional control [23,35-37]. By inspecting the RVD sequences of PthA2 and PthA4 (Figure 1B), we presumed that they would target common host genes. Consistent with this observation, we found that the transient expression of PthA2 or PthA4 resulted in the up-regulation of a similar group of genes that are functionally related to both defense and disease development (Additional file 2). The genes associated with canker development that were most strongly induced by both PthA2 and PthA4, encode cell-wall synthesis and remodeling enzymes, including a glycosyl transferase ortholog of *upa15* (CV709535) that is readily up-regulated by AvrBs3 in pepper plants [10]. In addition, the defense response induced genes encode chitinases, PR proteins and an ACC synthase, which are also up-regulated by Xc in the presence of Ch, thus providing initial evidence that they might be functional targets of PthA2 and PthA4 (Additional files 1 and 2). Notably, numerous genes up-regulated in response to PthA2 or PthA4 are functionally related to auxin and gibberellin synthesis and signaling, cell division and growth, and defense responses (Additional file 2). In particular, we noticed a strong PthA4-dependent induction of genes encoding cell division and expansion proteins including kinesins, tubulins, histones, ribosomal proteins, and orthologs of *dem* (*defective embryo and meristems*) and LOB (lateral organ boundary) (Additional file 2). Besides, the transcriptional profile of epicotyls expressing PthA4 is remarkably similar to that of citrus leaves infiltrated with Xc 48 h post-inoculation [26], as shown by the up-regulation of a large number of genes related to auxin synthesis, mobilization, and signaling, including two homologs of the auxin influx carrier protein AUX1 (CV706455, CX053885) [38-40] (see Venn diagram in Additional file 2). In contrast, epicotyls expressing PthA2 showed the up-regulation of many genes implicated in cell-wall remodeling and gibberellin synthesis and signaling, including orthologs of *upa6* and *upa7* (alpha-expansins), and *upa22* (GA-like protein), respectively, which are putative targets of AvrBs3 in pepper [10,31] (Additional file 2). PthA2 also induced several genes encoding retrotransposons (Additional file 2), which were also reported as off-targets of AvrXa7 in rice [25].

**Figure 2:**
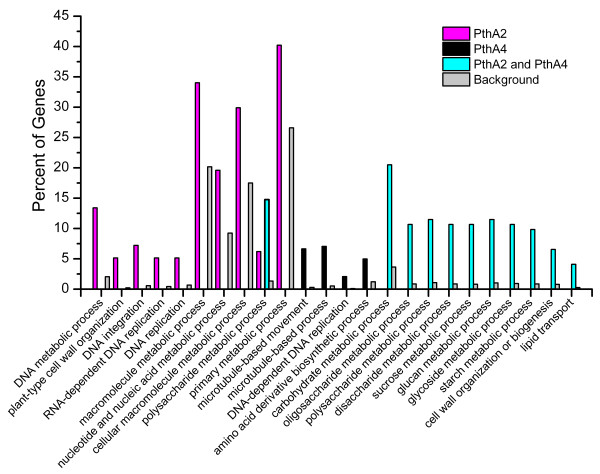
**GO enrichment analysis for PthA2 and/or PthA4-regulated genes.** GO enrichment analysis performed with agriGO [42], showing that PthA2 and PthA4 modulate several genes in sweet orange associated with canker development. Despite the fact that PthA2 and PthA4 regulate distinct, but related sets of genes implicated in cell-wall remodeling, cell division and growth, they both altered group of genes involved in carbohydrate metabolism and cell-wall organization and biogenesis. The identity of the enriched genes can be found in Additional file 2.

Gene onthology (GO) enrichment analysis showed that while PthA2 modulate several genes categorized in cell-wall organization and RNA-dependent DNA replication and integration, PthA4 affected the expression of genes grouped under the microtubule-based movement and cell growth category. In addition, the GO analysis showed that PthA2 and PthA4 commonly regulate a large number of genes involved in carbohydrate (sucrose, glucan and glycoside) metabolism, and cell-wall organization and biogenesis (Figure 2 and Additional file 2). These evidences support the functional convergence between Xc TAL effectors, and is in agreement with recent reports of *X. oryzae* TAL effectors [24,25]. In addition, the common targeting of PthA2 and PthA4 is a strong indicator that they may have additive or even synergistic roles for activation of host genes required for citrus canker susceptibility.

### Transcriptional changes in sweet orange triggered by PthC1, a TAL effector of *X. aurantifolii*

We have shown previously that XaC elicit an HR-type response in sweet orange leaves, which is characterized by the up-regulation of multiple defense-related genes [26]. To examine whether XaC TAL effectors are involved in the host transcriptional defense response, we cloned two TAL effectors from XaC strain ICMP 8354, designated PthC1 and PthC2. The essential differences between XaC TAL effectors occur in the number of repeat domains and the nature of the repeat variable diresidues (RVDs), which altogether form the DNA-binding domain of the protein [12,13]. PthC1 has 18 RVDs or 17.5 repeat domains, and PthC2 is shorter with only 15 RVDs or 14.5 repeat domains (Figure 1B). Despite the overall structural similarities with PthA“s” of Xc, PthC1 and PthC2 are phylogenetically more closely related to PthB“s” and PthC“s” from other pathotype B and C strains, respectively (Figure 1C), that are distinguished by their limited host range [4,21]. In terms of the RVD composition, PthC1 appears also more similar to PthB and PthC of other citrus canker strains reported to induce weak disease symptoms or hypersensitive response in sweet oranges [4,21]. Despite the fact that PthC from a group C strain was not required for the HR elicited on grapefruit [4], we decided to test whether PthC1 induce the expression of defense-related genes in sweet orange, as we observed during XaC infection [26]. Therefore, we transfected PthC1 in sweet orange epicotyls and compared the transcriptional changes relative to epicotyls expressing PthA2, PthA4, or the GUS gene as reference (Figure 1A). We found that PthC1 elicit not only a different but also an opposite effect of PthA2 and PthA4, because its expression resulted in a general down regulation of genes involved in auxin and gibberellin synthesis and signaling, cell-wall remodeling, cell division, and defense responses (Additional file 3). The fact that auxin and gibberellin promote initial canker pustule formations [27] indicate that PthC1, in contrast to PthA2 and PthA4, do not contribute to canker symptoms in sweet orange. Nevertheless, we found Aux/IAA and bZip orthologous genes repressed by PthC1 (CV713157, CV704184, CK701644, CN182471), which function as negative regulators of the auxin and gibberellin signaling pathways affecting plant growth and development [43-45]. In addition, the down-regulation of genes encoding GH3-like enzymes (CF837666, CF837443) [46] and indole-3-acetic acid amido synthase (CV714093) suggest that PthC1 operates to increase the active pools of free auxin. On the other hand, we found no obvious HR-like executor among the genes up-regulated by PthC1 in sweet orange epicotyls (Additional file 3), although an AP2-domain transcription factor orthologous of the tomato Pti4 [47] might be an interesting candidate (Additional file 3).

Together, these data show that PthC1, in contrast to PthA2 and PthA4, regulate a different set of genes in sweet orange. Although these genes may not significantly favor host susceptibility, they do not appear to be elicitors of an HR response either. This idea is consistent with the observation that a knockout mutation of *pthC* in a group C strain resulted in loss of pathogenicity on lime, but still triggers the HR response on grapefruit [4].

### Computational prediction of EBEs for PthA and PthC in citrus genomes

The public release of the *Citrus sinensis* and *Citrus clementina* genomes together with the TAL effector code of DNA binding [12,13], provides a suitable model to investigate *in silico* the TAL effector-targeted genes in citrus. Because the computational tools available for TAL effector targets prediction do not yet support analyses of the citrus genomes [24,25,48], we designed a position weight matrix based on TAL effector-DNA association frequencies to predict and score EBEs for Xc and XaC TAL effectors in citrus gene promoters (Additional file 4). Putative EBEs for the four PthA“s” and the 2 PthC“s” of strains Xc 306 and XaC ICMP 8354, respectively, were identified in nearly one thousand promoters. We then selected the top one hundred best scoring *C. sinensis* promoters ranging from 8.5 to 17.4 for further analysis (Additional file 5). In order to test the performance of our computational matrix analysis, we used the target finder function of the TAL Effector-Nucleotide Targeter 2.0 (TALE-NT) tools [48] to search for PthA“s” and PthC“s” EBEs in the top twenty best scoring promoters. We retrieved virtually the same EBE predictions with equivalent score values [data not shown], indicating a similar achievement between our prediction method and the TALE-NT tools.

Next, we functionally categorized our candidates based on sequence homology to plant, yeast, or animal gene orthologs with known biological function (Additional file 5). In addition to genes implicated in auxin and gibberellin synthesis and signaling, and in cell-wall remodeling, we found a substantial number of genes involved in cell division and morphogenesis, transcription regulation and defense (Additional file 5). Although most of our best scoring promoters do not correspond with the genes identified in our microarray analyses (see below), these data seem to be meaningful because the predicted candidates belong to the same functional categories of those up-regulated in citrus epicotyls in response to PthA/PthC expression or in Xc-infiltrated leaves in the presence of Ch (Additional files 1, 2, 3). Remarkably, we predicted two PthC1 targets, orange1.1g035902m.g and orange1.1g035488m.g (Additional file 5), with a strong similarity to the *Bs3* gene of pepper, which is an HR executor transcriptionally activated by AvrBs3 of *X. vesicatoria*[7]. We also found that several PthA“s” putative targets are involved in abscisic acid (ABA) synthesis, signaling and response, particularly for PthA1 (Additional file 5). Interestingly, some of these genes, including an ABA8-hydroxilase (orange1.1g012199m.g), ABI3 (orange1.1g038867m.g) and ABI3-interacting protein-1 (orange1.1g044737m.g), also participate in the cross-talk between auxin and gibberellin, and in plant growth and development [49-51] (Additional file 5).

### Experimental validation of putative gene targets of PthA and PthC effectors

Although computational identification of TAL effector-targeted genes have been recently conducted for TAL effectors of *X. oryzae* and Xc in their corresponding host genomes, the studies for Xc are largely deficient on experimental validation for novel candidate targets [24,25]. Nevertheless, the combination of *in silico* predictions with gene expression data, demonstrated to be a suitable strategy to identify new biologically relevant TAL effector targets [52]. Thus, in order to verify our *in silico* target predictions, we used the whole set of microarray data in our hands to search for experimental evidence of gene regulation of our predicted TAL effector targets. We found that nearly 20% and 3% of the computational-predicted targets were up and down-regulated, respectively, indicating that TAL effectors not only induce but may ultimately repress the expression of host predicted targets (Additional files 1, 2, 3 and 5). Using a cross-check criteria we were able to select targets that 1) are differentially expressed in epicotyls expressing the corresponding TAL effector, 2) are also differentially expressed after infiltration of Xc in the presence of Ch, and 3) are functionally associated with the mechanisms of canker development or defense response (Table 1).

**Table 1:** **Predicted TAL effector targeted genes in *C. sinensis *** **that are transcriptionally regulated by Xc in the presence of Ch or, by the heterologous expression of the TAL effector**

**Microarray data (fold change)**	**Sweet orange genes with**	**Homologous gene description**	**Functional category**	**References**
	**PthA2 binding sites**			
PthA2 x GUS (4.0)	orange1.1g032466m.g	Pepper UPA22 (UPA22)	GA response	[10,31]
	orange1.1g031880m.g	Tobacco rac-like GTPase 1 (RAC)	Auxin response	[53]
	orange1.1g001197m.g	Rat transcription activator BRG1 (BRG1)	Cell growth	[54]
PthA2 x GUS (-3.1)	orange1.1g023431m.g	Xyloglucan endotransglucosylase (XET)	Cell growth	[55]
	orange1.1g040761m.g	Castor bean LOB domain protein (LOB2)	Defense	[56]
PthA2 x GUS (-3.1)	orange1.1g037640m.g	Tobacco UDP-glucosyltransferase (UDPGT)	Defense	[57]
	**PthA4 binding sites**			
Xc + Ch x Ch (3.8)	orange1.1g024897m.g	Tobacco 14-3-3 protein (14-3-3)	Cell growth	[58-60]
PthA4 x GUS (11.4)	orange1.1g017949m.g	*Citrus limetta* dioxygenase (DIOX)	Cell growth	[61,62]
PthA4 x GUS (5.2)	orange1.1g018649m.g	Tobacco cysteine proteinase (CP)	Defense	[63,64]
	orange1.1g037138m.g	*C. trifoliata* NBS-LRR protein (CCNBS1)	Defense	[65]
	**PthC1 binding sites**			
	orange1.1g041266m.g	Tomato self pruning-interacting protein 1 (SIP1)	Cell growth	[66]
	orange1.1g010756m.g	Potato Ca^+2^-dependent protein kinase (CDPK)	Cell growth	[67]
	orange1.1g039072m.g	Potato CC-NBS-LRR protein (CCNBS2)	Defense	[68]
	orange1.1g042296m.g	Sunflower CC-NBS-LRR protein (CCNBS3)	Defense	[69]
	**Multiple EBEs**			
	**PthA2, PthA4, PthC1**			
	orange1.1g046669m.g	Tobacco Avr9/Cf9 elicited protein 146 (AE146)	Defense	[70]
	**PthA4, PthC1**			
Xc + Ch x Ch (8.8)	orange1.1g026556m.g	Aspen LOB domain 1 (LOB1)	Cell growth	[71]

**Figure 3:**
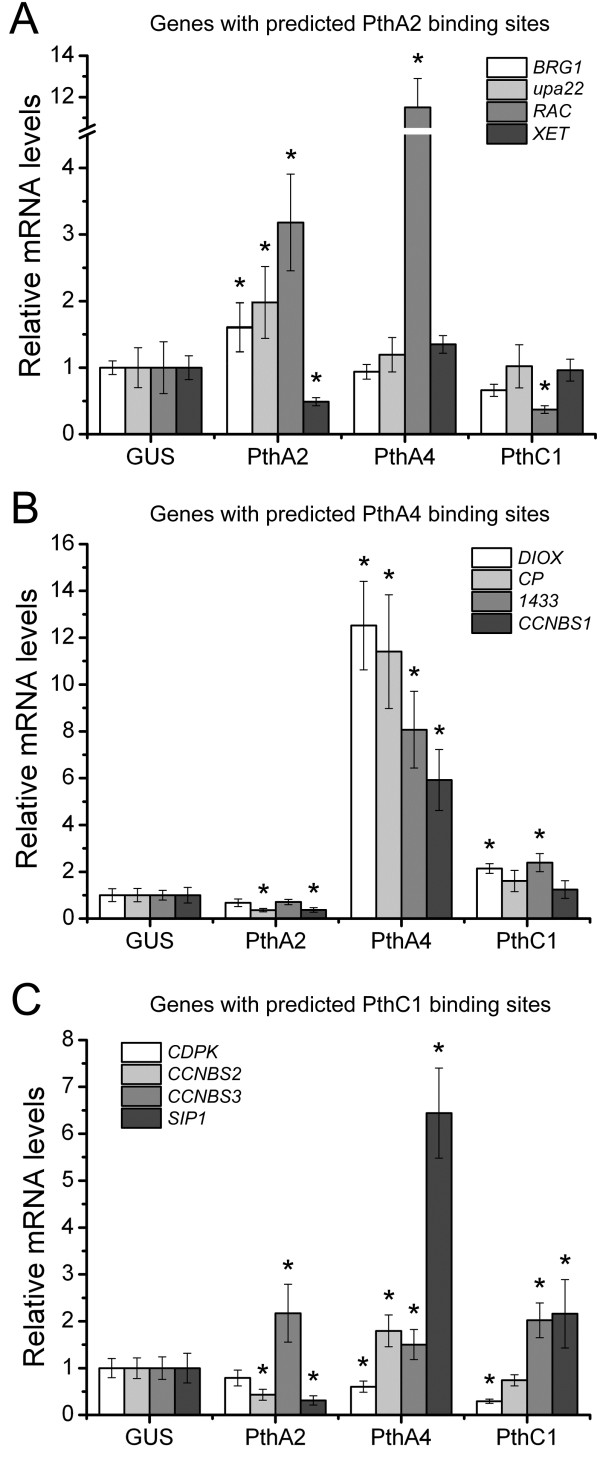
**Gene expression levels of predicted targets of PthA2, PthA4 and PthC1 in epicotyls transfected with the corresponding TAL effector. (A)** Quantitative RT-PCR (qPCR) of four sweet orange genes with best-scoring effector-binding elements (EBEs) predicted for PthA2 in their promoters. **(B)** qPCR of four sweet orange genes with best-scoring EBEs predicted for PthA4 in their promoters. Predicted PthA4 target genes are significantly and predominantly up-regulated by PthA4 expression. **(C)** qPCR of four sweet orange genes with best-scoring EBEs predicted for PthC1 of XaC in their promoters. The expression levels are the mean of three independent biological replicates. The error bars denote standard deviations whereas asterisks indicate statistically significant differences (p < 0.05) in the mRNA levels in epicotyls expressing the TAL effectors relative to GUS.

To verify these data, we first confirmed the TAL effector protein accumulation in epicotyls transiently expressing *pthA2*, *pthA4* or *pthC1* (Figure 1A), and subsequently examined the mRNA levels of the predicted targets by quantitative RT-PCR on the transgenic tissues. Totally, sixteen genes implicated in symptom development or defense response, were selected for target validation (Table 1). These candidates were differentially expressed in response to Xc infiltration and also after TAL effector expression in epicotyls, and encoded at least one predicted EBE for the corresponding TAL effector in their promoters. Initially, we tested the expression of four putative PthA2 targets, including an ortholog of the pepper *upa22*, which encodes a xyloglucan endotransglucosylase (*XET*), a RAC-GTPase gene (*RAC*), and an ortholog of the rat BRG1, implicated in cell wall strengthening, auxin response, and tumor development, respectively (Table 1). Consistent with the microarray data (Table 1), the citrus *upa22* and the *BRG1* genes were slightly but preferentially up-regulated by PthA2 compared to PthA4, PthC1 or GUS in epicotyls; in contrast, *XET* was significantly down-regulated in response to PthA2 (Figure 3A). The *RAC* gene, which was induced in epicotyls expressing PthA2 and PthA4, also appeared down-regulated in tissues expressing PthC1 (Figure 3A). Despite the strong induction of *RAC* by PthA4 (Figure 3A), we found no good scoring candidate EBEs for PthA4 in the *RAC* promoter. Presumably, this is part of false negative predictions that account for the current limitations of computational analyses of TAL effector targets [17,52,72,73].

To test the PthA4 candidate targets, we selected 4 genes encoding orthologous of a tobacco 14-3-3 protein, a two-domain dioxygenase (DIOX), a cysteine protease (CP), and a CC-NBS-LRR protein (CCNBS1), which play roles in gibberellin synthesis, cell elongation and defense (Table 1). Consistent with the microarray data (Table 1), we found that the four selected genes were strongly (more than 6 fold-change) up-regulated in epicotyls expressing PthA4, which support the computational prediction of best scoring targets of PthA4 (Figure 3B). Although *14-3-3* and *DIOX* were induced moderately (between 2 to 3 fold-change) in epicotyls expressing PthC1, no EBEs for PthC1 were identified in these targets.

We also tested the PthC1 predictions using 4 selected candidate genes, including the orthologs of a potato Ca^+2^-dependent protein kinase (*CDPK*) and tomato self-pruning interacting protein 1 (*SIP1*), and two CC-NBS-LRR resistance genes (*CCNBS2* and *3*) (Table 1). An ortholog of the pepper *Bs3* gene (orange1.1g035488m.g) was also tested; however, we were not able to detect this gene by qPCR analysis, even using large amounts of cDNA input. We found that *SIP1* and *CCNBS3* genes were significantly induced in response to PthC1 expression relative to GUS, although *CCNBS3* was also up-regulated by PthA2 and 4 (Figure 3C). In addition, *SIP1* and *CCNBS2* appeared also induced in response to PthA4 expression. Interestingly, we found an EBE for PthA4 in the *SIP1* promoter with a score value of 6.2 that explains the observation. In contrast, *CDPK* was 3-fold repressed by PthC1 (Figure 3C).

Altogether, these results partially confirm the TAL effector targets prediction but also suggest that more than one TAL effector might induce the same gene, and that TAL effectors can ultimately cause transcriptional repression of targets, although we have not tested whether this is a direct or indirect (secondary) effect of the protein.

**Figure 4:**
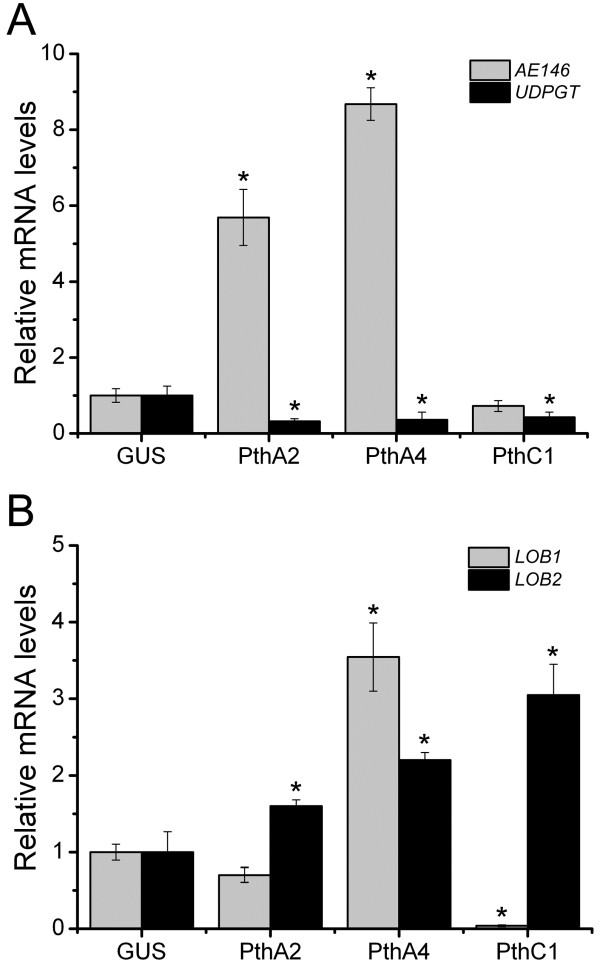
**Expression levels of possible common targets of PthA2, PthA4 and/or PthC1. (A)** Expression levels of the Avr9/Cf9-elicited 146 (*AE146*) and *UDPGT* genes in response to TAL effector expression in citrus epicotyls. Although binding sites for PthA2, PthA4 and PthC1 were identified in the *AE146* gene promoter, *AE146* is strongly induced by PthA2 and 4 only. The UDPGT gene, which was down-regulated by PthA2 in microarray experiments, and has a predicted PthA2-binding site in its promoter, is repressed by all three effector proteins. **(B)** Expression levels of two citrus *LOB* genes (*LOB1* and *LOB2*) in response to TAL effector expression in citrus epicotyls. LOB1, identified as a target of PthA4 and PthC1 was preferentially modulated by these effectors, whereas *LOB2*, identified as a PthA2 target by EBE prediction is apparently up-regulated by all three effectors. The expression levels are the mean of three independent biological replicates. The error bars denote standard deviations whereas asterisks indicate statistically significant differences (p < 0.05) in the mRNA levels in epicotyls expressing the TAL effectors relative to GUS.

To further investigate these observations, we evaluated the expression of a predicted PthA2 target, a phenylpropanoid:UDP-glucosyltransferase (*UDPGT*), which was nearly 3-folds down-regulated in PthA2-expressing epicotyls (Table 1). We also tested a predicted target of PthA2, PthA4 and PthC1 encoding an Avr9/Cf9-elicited protein 146 homolog, *AE146*, as well as two Lateral Organ Boundaries genes, *LOB1* and *LOB2*, indentified as targets of PthA4/PthC1 and PthA2, respectively (Table 1). A previous report predicted that the citrus *LOB1* was targeted by PthA4 [24]. We found that *AE146* is induced in response to PthA2 and PthA4 expression, but in contrast, PthA2, PthA4 and PthC1, down-regulate the expression of *UDPGT* (Figure 4A). Consistent with our microarray data (Table 1), *LOB1* was predominantly induced by PthA4, but repressed by PthC1, while all the three TAL effectors tested induce the expression of *LOB2* (Figure 4B).

**Figure 5:**
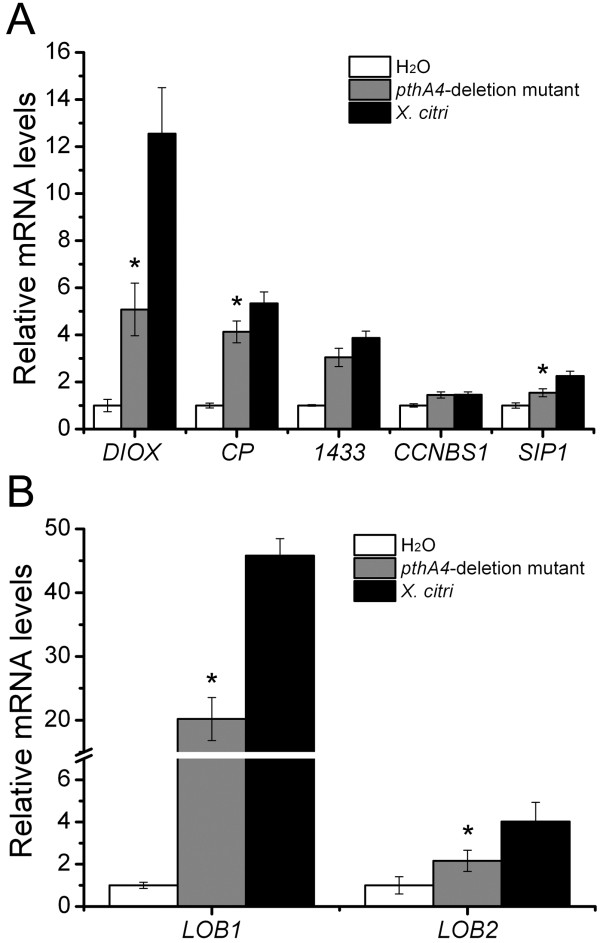
**Expression levels of potential targets of PthA4.** Expression levels of *DIOX*, *CP*, *14-3-3* (1433), *CCNBS1* and *SIP1***(A)**, or *LOB1* and *LOB2***(B)**, in sweet orange leaves infiltrated with Xc or its mutant derivative lacking *pthA4* (*pthA4*-deletion mutant), 72 h post-inoculation, relative to water-infiltrated leaves. All target genes were induced by Xc and *pthA4*-deletion mutant, relative to water infiltration; however, the expression levels of most genes, including *DIOX*, *SIP1*, *LOB1* and *LOB2*, were significantly lower in the leaves infiltrated with the *pthA4* mutant, suggesting a role of PthA4 in gene activation. The expression levels are the mean of three independent biological replicates. The error bars denote standard deviations whereas asterisks indicate statistically significant differences (p < 0.05) between the mRNA levels found in the leaves infiltrated with the *pthA4* mutant, relative to the wild type *X. citri*.

We also used a *pthA4* knockout derivative of Xc, which is not pathogenic in sweet orange neither it induces hyperplasic lesions [23], to analyze the expression levels of candidate PthA4 targets. We found that three of the predicted PthA4 targets, *DIOX*, *CP* and *14-3-3*, are induced at higher levels in leaves infiltrated with the wild type Xc strain relative to the *pthA4*-deletion mutant; however, these genes were also up-regulated by the *pthA4* mutant relative to water infiltration, which indicates that these genes present a more complex mechanism of induction where PthA4 is not absolutely required (Figure 5A).

We also evaluated the expression of two PthA2 predicted targets *RAC* and *LOB2*, and two PthC1 targets *LOB1* and *SIP1*, which were highly up-regulated in epicotyls expressing PthA4 (Figure 3A and C). Although *RAC* expression was under the level of detection by quantitative PCR (not shown), we found that the transcript accumulation of *SIP1* was lower in response to *pthA4*-deletion mutant relative to leaves infiltrated with the wild type Xc (Figure 5A). Similarly, the expression levels of *LOB1* and *LOB2* were significantly lower in leaves infiltrated with the *pthA4*-deletion mutant (Figure 5B). Nevertheless, *LOB1*, *LOB2* and *SIP1* were also up-regulated in leaves infiltrated with the *pthA4*-deletion mutant relative to water-infiltrated leaves, suggesting an alternative mechanism of target induction, potentially by other TAL effectors that our analysis failed to predict.

Taken together these results confirm our previous observations, and point out to *LOB1*, *LOB2, SIP1, CP* and *DIOX* as primary targets of Xc and XaC TAL effectors, in particular of PthA4 (Figure 5).

### PthA and PthC EBEs overlap with or localize close to TATA box elements in citrus promoters

**Figure 6:**
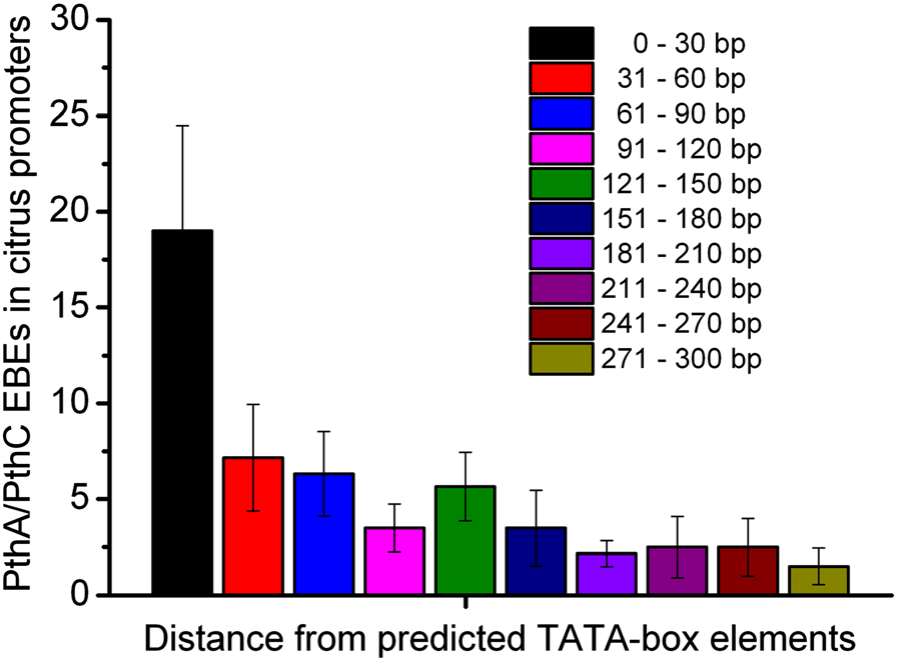
**Distribution of average distance of predicted EBEs relative to TATA-like elements in citrus promoters.** Possible overlap between TATA box and PthA/PthC-binding site positions in the citrus promoters predicted as targets of PthA/PthC proteins. The great majority of PthA and PthC-binding sites overlap with, or are located within 30 bp of the predicted TATA-box elements in the corresponding citrus promoters.

Including AvrBs3, several TAL effectors bind to EBEs that overlap with TATA-like sequences [6-8,29]. In some cases this causes a downstream shift of the transcriptional start site in the targeted gene, suggesting that TAL effectors might have a similar function of TATA-binding proteins [6-10]. Here, we found that approximately 73% of the predicted EBEs for PthA“s” and PthC“s” localize between 16 and 300 bp upstream the translation start codon of the genes (Additional file 5). This observation and a recent study in rice suggesting that TAL effectors of *X. oryzae* are predicted to bind within 300 bp upstream the start codon and frequently overlap with TATA-box elements of the promoter [24], prompted us to evaluate whether the overlap or close proximity between TATA boxes and EBE positions, also occurs for TAL effectors of Xc and XaC in citrus promoters. We found that most of the EBEs predicted for PthA“s” and PthC“s” show a tendency to overlap with, or localize within 30 bp of putativeTATA-box elements (Figure 6 and Additional file 5). For instance, in five of the candidate targets (*LOB1*, *LOB2*, *AE146, CCNBS1* and *CDPK*) the EBEs overlapped with a putative TATA box (Additional file 5). Thus, our data suggest that despite the RVD variations among these TAL effectors, they are likely to have an evolutionary selection pressure towards the targeting of TATA-rich regions of host sequences that are critical for the transcriptional regulation.

## Discussion

Despite the fact that many TAL effectors targets and their molecular functions have been coming to light in recent years [3,6-11,74,75], citrus genes directly activated by PthA“s” or PthC“s” effectors, and their role in canker development or host defense against *Xanthomonas spp.*, remain poorly characterized. In this study, we have identified a number of genes putatively targeted by PthA“s” and PthC“s” in sweet orange, and shown that most of them impinge on disease and/or host defense responses. We also observed that PthA2 and PthA4, yet bearing distinct RVDs composition (Figure 1B), exhibit functional convergence, in particular to regulate genes of the auxin and gibberellin synthesis and response pathways, as well as their downstream signaling cascade genes like those for cell wall remodeling, cell division and expansion. In citrus, targeting these hormonal pathways appear to be the headline of canker-causing *Xanthomonas spp.* since endogenous auxin and gibberellin are required for initial canker formation in leaves infiltrated with the pathogen, and the exogenous application of the hormones transcriptionally regulate specific genes also induced by Xc during the onset of infection [26,27]. How these hormones contribute to the canker disease needs further exploration, but it is clear their relationship with the canker symptoms, hypertrophy and hyperplasia of mesophyll cells [2], which generate enough internal pressure to prompt the epidermal rupture and facilitate pathogen release to the leaf surface for disease propagation [21,27]. Besides, auxin also increase the host susceptibility on other plant-pathogen interactions [76-79], pointing it out as a conserved mechanism of biotrophic microbes to elicit cell wall softening to e.g., leak nutrients from the host cell, and/or to improve effectors translocation that promote host susceptibility and bacterial fitness. Overall, it is tempting to speculate that, in the case of the citrus canker pathogens, manipulating the auxin signaling pathway via TAL effector-targeting would be an effective way to disarm host defense responses for bacterial survival and pathogenicity. Notably, many of the AvrBs3 up-regulated (*UPA*) genes are also auxin-responsive and some of them including *upa7*, *upa15*, *upa17* and *upa20*, are known to enhance cell hypertrophy and the synthesis of cell-wall polymers [8,10,31].

These data reflect on recent studies suggesting that the redundancy and convergence in TAL effector repertories occurring within and across *X. oryzae* strains, respectively, may be a conserved feature of TAL effector evolution as a consequence of an arms race of the host-bacteria interaction [24,25]. The biological meaning of such functional redundancy of TAL effectors in strain 306, particularly between PthA2 and PthA4, denote a cooperative, or eventually a synergistic role of these effectors as transcriptional activators (Figures 3, 4, 5, Additional files 2 and 5). In addition, it is interesting to note that PthA2 physically interacts with PthA4, and that both proteins bind independently to the C-terminal domain of RNA Pol II [37]. Hence, TAL effector-mediated transcriptional regulation in citrus appears to be further influenced by the multiple protein-protein interactions occurring at the assembly locus of RNA Pol II. According with this assumption, the up-regulation of *LOB1* and *LOB2* in epicotyls expressing PthA2 or PthA4 (Figure 4B), and in sweet orange leaves infiltrated with Xc or its *pthA4* mutant derivative (Figure 5B), emerges as an evident example of a redundant targeting of a presumably major gene for canker susceptibility. We also predicted another gene encoding a LOB-domain protein targeted by PthA3, orange1.1g048558m.g (Additional file 5). Interestingly, *LOB* genes have been associated to auxin signaling pathways, including cell growth control and organ development [56,71,80]. From an evolutionary point of view, it seems advantageous for a single Xc strain to target a family of potential susceptibility genes, i.e. *LOB* genes with different TAL effectors, which function cooperatively or synergistically, because it will assure the induction of the critical set of genes involved in disease symptoms development. The observation is convincing since it implies a mechanism that the pathogen uses to overcome potential natural polymorphisms in TAL effector targeted promoters, in particular those occurring in susceptibility genes among different host varieties [6].

Beyond the functional redundancy of Xc TAL effectors, we found that PthA4 predominantly induce genes encoding ribosomal proteins and proteins of cell division and growth, like kinesins, tubulins and histones (Additional file 2). This is noteworthy because PthA4 also physically interacts with the citrus MAF1 protein (CsMAF1), which is a negative regulator of RNA Pol III that controls ribosome biogenesis and suppresses canker symptoms [23,36]. Thus, the specific PthA4-induction of ribosomal protein genes appears to be a causal feedback mechanism of ribosome biogenesis that is required for cell division and growth. On the other hand, although PthA2 does not interact with CsMAF1, it binds to and inhibits the prolyl-isomerase activity of the citrus cyclophilin CsCYP, a putative negative regulator of the RNA Pol II complex [23,35,37,81]. Therefore, it remains to be determined how these protein specific interactions between TAL effectors and components of RNA Pol II and III complexes affect the outcome of the host expression profiles in response to bacterial infection.

In contrast to PthA“s”-induced genes, epicotyls expressing PthC1 showed a general down-regulation of auxin and gibberellin response genes suggesting that this TAL effector is not an elicitor of susceptibility. For instance, *LOB1* that is induced by PthA4, appears significantly down-regulated by PthC1 (Figure 4B), which is consistent with the PthC1-mediated repression of auxin response genes, since LOB proteins have also been implicated in auxin signaling [80,82]. However, it remains to be determined whether the general down-regulation of the auxin responsive genes by PthC1 in sweet orange is the major cause for the lack of host susceptibility (i.e. no hyperplasic lesions) after infiltration of sweet orange with XaC [26].

In pepper plants ecotype ECW-30, the *Bs3* resistance gene induced by the *X. vesicatoria* TAL effector AvrBs3, executes an HR response after bacterial infiltration [7]. Our computational analyses have predicted EBEs of PthC1 in the promoters of two sweet orange genes orthologous to *Bs3*, orange1.1g035488m.g and orange1.1g035902m.g (Additional file 5); however, we were not able to detect their expression in response to PthC1 or after XaC infiltration (Additional file 3), and neither were them detected by any other gene expression analyses we conducted before i.e., differential display PCR and suppression subtractive hybridization [26]. Besides, though the results discussed above indicate that XaC cannot induce host susceptibility, we do not yet discard the possibility that an HR-like elicitor is mediating the sweet orange defense against XaC.

It has been verified and predicted that nodulin and flavone-3-hydroxylase (F3H) genes, respectively, represent common targets of *Xanthomonas* ssp. TAL effectors [11,24,25]. Here, we found four nodulin-related genes, which are significantly up-regulated in sweet orange leaves infiltrated with Xc in the presence of Ch, DN620509, or in epicotyls expressing PthA2, CX675781, and PthA4, DN958192 and CV710110. However, we were not able to predict EBEs for PthA“s” in their promoters suggesting a potential gap for false negative predictions of computational analyses, or eventually indicating that their induction is consequential of other primary targets of the TAL effectors. On the other hand, we predicted EBEs of PthA and/or PthC in the promoters of the nodulin-related genes orange1.1g007766m.g and orange1.1g042021m.g, but none of these genes were up-regulated in our microarray dataset (Additional files 1, 2 and 5).

One of the predicted targets of PthA4 is the citrus dioxygenase gene *DIOX* (Table 1, Figures 3B and 5A, Additional file 5) that is similar to the rice gene *F3H*, also a predicted target of TAL effectors from *X. oryzae* strains [25]. Although the F3H was linked to flavonoid biosynthesis [25], both *F3H* and *DIOX* are similar to Gibberellin-20-Oxidases (GA20ox), a group of enzymes that catalyze gibberellin biosynthesis [83]. Interestingly, as observed for the *LOB* genes, a citrus gene encoding a putative GA20 oxidase (orange1.1g019643m.g) was identified as a target of PthA1, PthA2 and PthA3 (Additional file 5). This protein has the same domain architecture found in DIOX, which suggests they are functional orthologs. In addition, both PthA4 and PthC1 transactivate a regulator of gibberellin synthesis namely *14-3-3*, and a *SIP1* gene in epicotyls (Figure 3) whose protein products interact with a SELF-PRUNING factor involved in the control plant architecture and flowering of tomato [58,66,84]. These evidences together support the susceptibility effect of inducing gibberellin genes during the process of infection.

Another outcome of our study is the proximity of the predicted EBEs relative to putative TATA boxes of promoter regions (Figure 6). This is in line with the fact that EBEs for *X. oryzae* TAL effectors have been also identified close to TATA elements in rice promoters [6]. Similarly, AvrBs3 binding sites are found within 100 bp upstream of the transcription start site [10], raising the idea that TAL effectors might functionally replace general TATA-binding factors, as recently proposed [24]. In addition, our data indicates that TAL effectors can function as transcriptional repressors, in particular PthC1, as discussed above (Additional files 2 and 3; Figures 3 and 4).

## Conclusion

In conclusion, we have identified candidate targets of PthA“s” and PthC“s” in citrus that will not only strengthen our understanding on canker symptoms formation, but also provide novel information about host susceptibility or defenses against *Xanthomonas* pathogens, which will assist in the selection and generation of canker resistant plants.

## Methods

### Bacterial strains, plasmids and growth conditions

PthA2 and PthA4 were amplified from the Xc strain 306 [85] and cloned into pET28a and pBI121 for bacteria and plant expression, as previously described [35]. Plasmids were introduced into *E. coli* strain DH5alpha and/or *Agrobacterium tumefaciens* strain EHA105 by electroporation. *E. coli* cells were incubated at 37°C in Luria-Bertani (LB) medium, whereas Xc and *A. tumefaciens* were grown in LB without NaCl at 28°C and in YEP (Bacto peptone 10 g/l, NaCl 5 g/l, Yeast extract 10 g/l, and Agar 15 g/l) at 30°C, respectively [86]. Bacterial cultures were grown at different time periods until they reached the desired optical densities. Antibiotics were added to the media in the following final concentrations: ampicillin, 100 μg/ml; kanamycin, 50 μg/ml; rifampicin, 50 μg/ml; streptomycin, 25 μg/ml.

### Plant material, bacterial infiltration

Six-month-old plants of sweet orange “Pera” were obtained from certified nurseries and kept in a growth room at 25–28°C with a 14 h light photoperiod. For plant infiltration, Xc strains were inoculated from single colony plates and grown over night at 28°C in liquid LB without NaCl. Cells colonies were suspended in sterile water to an optical density at 600 nm (OD_600_) of 0.1 (nearly 10^6^ CFU/ml). Leaves were infiltrated with bacterial suspensions in water or, in 50 μM cycloheximide (Ch) (Sigma-Aldrich, USA). Water and Ch only were independently infiltrated as mock controls. For quantitative PCR (qPCR) assays, fully expanded “Pera” leaves were infiltrated with a water suspension (OD_600_ = 0.1) of wild type Xc strain 306, or its *pthA4*-deletion mutant derivative [23].

### Cloning TAL effectors from *X. aurantifolii*

PthC genes were PCR amplified using total DNA extracted from the XaC strain ICMP 8435 [26] as template. Primers targeting conserved 5’ and 3’ regions were designed based on the four available Xc *pthA* genes (5’-CATATGGATCCCATTCGTTCG-3’ and 5’-GAATTCTCACTGAGGCAATAGCTC-3’). PCR products were amplified using the Pfu turbo DNA polymerase in a 50 μL reaction, following the supplier’s instructions (Stratagene, USA), with an annealing temperature of 52°C and extension time of 4 min. PCR products were gel-purified and cloned into of pET28a vector using the restriction sites *Nde*I/*EcoR*I. At least twenty independent clones were analyzed by restriction mapping with *Ssp*I enzyme, or by DNA sequencing and only two different variants designated PthC1 and PthC2 were identified. None of the PthA effectors of Xc isolate 306 were found in our screening of XaC TAL effectors sequences.

### Expression of *Xanthomonas* TAL effectors in citrus epicotyls

*Agrobacterium* strains transformed with pBI121 vector (bearing the *uidA* gene under the control of the CaMV 35S promoter) or its derivative carrying the TAL effectors *pthA2*, *pthA4* or *pthC1* in place of the *uidA* gene, were used to transform sweet orange. Epicotyls from young plantlets of *Citrus sinensis* ‘Hamlin’ were wounded, transversely sectioned and incubated at room temperature (21°C) for 15 minutes in a fresh suspension of *A. tumefaciens* (OD_600_ = 0.6) containing 100 μM acetosyringone. Co-cultivation assays were conducted on semi-solid 1x Murashige and Skoog medium supplemented with 25 g/l sucrose, vitamin cocktail (10 mg/l thiamine-HCl, 10 mg/l pyridoxine, 1 mg/l nicotinic acid, 0.4 mg/l glycine), 100 mg/l of *myo*-inositol and 0.2 mg/l of 2,4-dichlorophenoxyacetic acid (pH 5.8) during 72 h in dark [87]. Transformation efficiency was confirmed by western blot analyses and histochemical GUS assay prior to RNA isolation for microarray and qPCR analysis.

### Western blot detection of PthA/PthC expression

Citrus epicotyls transfected with the native pBI121 vector or, its derivatives carrying the effector genes *pthA2*, *pthA4* or *pthC1*, were grinded to homogeneity in SDS-PAGE sample buffer and resolved on a 10% SDS-polyacrylamide gel. The proteins were transferred onto PVDF membranes and probed with anti-PthA2 serum as previously described [35].

### GUS assay

The *A. tumefaciens* strain EHA105 transformed with the pBI121 vector carrying the reporter gene *uidA*, which encodes beta-D-glucuronidase (GUS), was transfected in citrus epicotyls as described previously and the transient expression of GUS was tested using colorimetric assay 72 h after bacteria-tissue co-cultivation [88].

### RNA isolation and microarray and RT-qPCR analysis

Messenger RNA (mRNA) was extracted from infiltrated leaves or from transformed epicotyls as described previously [26]. For microarray hybridization, approximately 0.6 μg of mRNA was used to synthesize cDNAs, which were subsequently used as template to generate the biotin-labeled complementary RNAs (cRNAs) using the One-Cycle target labeling assay (Affymetrix). GeneChips of citrus genome arrays (Affymetrix) were hybridized with cRNAs following standard instructions of Affymetrix kits. The hybridized arrays were rinsed, stained and scanned with an Affymetrix Genechip Scanner 3000–7G. Two CEL files per treatment, corresponding to biological replicates, were analyzed with the ArrayAssit software package (ArrayAssit x.5, Stratagene, USA) using the MAS5 algorithm.

Total RNA samples were prepared from sweet orange leaves infiltrated with Xc, its *pthA4*-deletion mutant, or water as control, 72 h after bacterial inoculation, using the Trizol method (Invitrogen, Carlsbad, CA) and subsequently treated with DNase I (Promega, Madison, WI). Nearly 10 μg of total RNA was reverse-transcribed using the Maxima First Strand cDNA Synthesis Kit (Fermentas) according to the supplier’s instructions, and used as template in real-time qPCR reactions conducted in 96-well plates. Primer sequences (Additional file 6) corresponding to the *Citrus sinensis* genes listed in Table 1 were designed using the Primer Express 2.0 software (ABI, Foster City, CA). Each 25-μL qPCR reaction was composed by 12.5 μL of SYBR green 2× master mix (ABI, Foster City, CA), 1 μL of forward and reverse primer mix (7.5 μM), 1 μL of cDNA and 10.5 μL of diethyl pyrocarbonate-treated water. qPCR amplifications were carried out using the 7500 system “Universal” cycle condition in an ABI Prism 7300 instrument (Applied Biosystems, Foster City, CA). The *Citrus sinensis* gene encoding a malate translocator was selected as internal control for normalization [86]. Total RNA from three different leaves were used in qPCR reactions as independent biological replicates, and three technical replicates for each biological sample were considered for statistical T-tests to determine the significant changes of gene expression across the treatments.

### *In silico* prediction of TAL effector binding sites

A position-specific weight matrix was designed based on the TAL code of associations frequencies between DNA bases and RVDs, and used to score putative effector binding elements (EBEs) of PthA1, PthA2, PthA3 and PthA4 of Xc strain 306, and PthC1 and PthC2 of XaC strain ICMP 8435, considering a thymidine (T) at position -1 of the EBE as preferential base [12,13]. The algorithm was used to perform systematic similarity searches within 1500 bp of promoter regions relative to the translation start site of genes annotated in the genomes of *C. clementina* (http://www.citrusgenomedb.org/species/clementina/genome1.0) and *C. sinensis* (http://www.citrusgenomedb.org/species/sinensis/genome1.0). Identification of orthologous relationships between these two species was based on nucleotide sequence similarities (BLASTn) and best bidirectional hit (BBH) method [89,90]. Reciprocal sequence similarity searches between *C. clementina* and *C. sinensis* genes were performed using the BLASTn algorithm [91], with an E-value cutoff of 1xE^-20^, and a homemade Perl script to parse both BLASTn outputs to identify the BBH BLAST relationships (Additional file 4). The search was performed using the MOODS algorithm [92], which provided a list of possible EBEs for the PthA/PthC TAL effectors with a corresponding score for each sequence. The p-value cutoff was set to 0.001 in order to reduce false positives prediction alignments.

The final set of *C. sinensis* promoters having one or more putative PthA/PthC binding sites (top 100 hits) was derived from a conserved region analysis between the *C. clementina* and *C. sinensis* orthologous promoters, an approach that is widely used to identify new regulatory motifs in many organisms [93-95]. Thus, only the citrus genes with PthA/PthC binding sites in both *C. sinensis* and *C. clementina* promoters were selected.

### In *silico* TATA-box prediction

The DNA sequences corresponding to the promoters of *C. sinensis* genes having potential EBEs (top hits) were submitted to the Plant TATA-box prediction server using the TSSP program [96] at the http://linux1.softberry.com/berry.phtml site.

### Availability of supporting data

The sequences of PthC1 and PthC2 were deposited in the GeneBank as ADI48327 and ADI48328 accessions, respectively. The microarray data files (Affymetrix CEL files) for all the experiments described here have been deposited to GEO under the superseries GSE51379.

## Competing interests

The authors declare that they have no competing financial interest.

## Authors’ contribution

Conceived and designed the experiments: ALAP, MFC, RAC and CEB, Performed the experiments: ALAP, MFC, VYA, MLPO, MND, JCS and CEB, Analyzed the data: ALAP, MFC, VYA, MND, RAC and CEB, Wrote the paper: RAC and CEB. All authors read and approved the final manuscript.

## Supplementary Material

Additional file 1 Microarray analyses of sweet orange leaves infiltrated with Xc in the presence or absence of cycloheximide (Ch), 8 h after bacterial inoculation. Click here for file

Additional file 2 Microarray analyses of sweet orange epicotyls expressing PthA2 or PthA4. Click here for file

Additional file 3 Microarray analyses of sweet orange epicotyls expressing PthC1. Click here for file

Additional file 4 Perl script developed to parse both BLASTn outputs to identify the best bidirectional hit (BBH) BLAST relationships. Click here for file

Additional file 5 **Top one hundred best scoring *C. sinensis *** **promoters with predicted EBEs for PthA and PthC proteins.** Click here for file

Additional file 6 List of primers used in the qPCR experiments. Click here for file
