# Supplementary material for: Identification of putative TAL effector targets of the citrus canker pathogens shows functional convergence underlying disease development and defense response
Source: BMC Genomics. 2014 Feb 25;15:157. doi: 10.1186/1471-2164-15-157 (PMC4028880; doi:10.1186/1471-2164-15-157)
Supplement: Additional file 4 — Perl script developed to parse both BLASTn outputs to identify the best bidirectional hit (BBH) BLAST relationships. [file 1471-2164-15-157-S4.DOCX]

**Additional file 4**

**Perl script developed to parse both BLASTn outputs to identify the best bidirectional hit (BBH) BLAST relationships**

#!usr/bin/perl -w

#To run use: perl get_bbh.pl Sinensis_vs_Clementina.BLASTn.out Clementina_vs_Sinensis.BLASTn.out

#Output: Orthologous gene pairs

use strict;

use Bio::SearchIO;

my $vr = new Bio::SearchIO(-format => 'blast',

-file => "<$ARGV[0]");

my $rv = new Bio::SearchIO(-format => 'blast',

-file => "<$ARGV[1]");

my %v2r=();

my %r2v=();

while( my $result = $vr->next_result ) {

my $query = $result->query_name;

if ($result->num_hits > 0) {

my $hit = $result->next_hit;

my $subject=$hit->name;

$v2r{$query}=$subject;

}

}

while( my $result = $rv->next_result ) {

my $query = $result->query_name;

if ($result->num_hits > 0) {

my $hit = $result->next_hit;

my $subject=$hit->name;

$r2v{$query}=$subject;

}

}

open(OP,">orthologues.txt");

foreach my $v(keys %v2r) {

if (defined $r2v{$v2r{$v}}) {

if ($v eq $r2v{$v2r{$v}}) {

print OP "$v\t$v2r{$v}\n";

}

}

}

close(OP);
